# Wuzi Yanzong Pill—Based on Network Pharmacology and *In Vivo* Evidence—Protects Against Spermatogenesis Disorder *via* the Regulation of the Apoptosis Pathway

**DOI:** 10.3389/fphar.2020.592827

**Published:** 2020-12-18

**Authors:** Wang-qiang Chen, Cai-fei Ding, Jia Yu, Chen-ye Wang, Ling-yi Wan, Hui-min Hu, Jian-xiong Ma

**Affiliations:** ^1^ Department of Reproductive Medicine, Zhejiang Provincial Integrated Chinese and Western Medicine Hospital, Hangzhou, China; ^2^ The Second Clinical Medical College, Zhejiang Chinese Medical University, Hangzhou, China; ^3^ Department of Andrology, Dongzhimen Hospital, Beijing University of Chinese Medicine, Beijing, China

**Keywords:** Wuzi Yanzong pill, spermatogenesis disorder, network pharmacology, bioactive compounds, hub targets, apoptosis pathway

## Abstract

The crisis of male infertility is an issue of human reproductive health worldwide. The Wuzi Yanzong pill (WZYZP) is a traditional Chinese medicine prescription that shows efficacy in kidney reinforcement and essence benefit to ameliorate male reproductive dysfunctions. However, the pharmacological mechanisms of the WZYZP on male infertility have not been investigated and clarified clearly. This study was designed to investigate the effects of the WZYZP on spermatogenesis disorder and explore its underlying pharmacological mechanisms. First, based on a network pharmacology study, 39 bioactive compounds and 40 targets of the WZYZP associated with spermatogenesis disorder were obtained, forming a tight compound-target network. Molecular docking tests showed tight docking of these compounds with predicted targeted proteins. The protein–protein interaction (PPI) network identified TP53, TNF, AKT1, Bcl-XL, Bcl-2, and IκBA as hub targets. The Kyoto Encyclopedia of Genes and Genomes pathway network and pathway-target-compound network revealed that the apoptosis pathway was enriched by multiple signaling pathways and multiple targets, including the hub targets. Subsequently, the chemical characterization of WZYZP was analyzed using liquid chromatography to quadrupole/time-of-flight mass spectrometry, and 40 compounds in positive ion mode and 41 compounds in negative ion mode in the WZYZP were identified. Furthermore, based on the prediction of a network pharmacology study, a rat model of spermatogenesis disorder was established to evaluate the curative role and underlying mechanisms of the WZYZP. The results showed that WZYZP treatment improved rat sperm quality and attenuated serum hormone levels, reversed histopathological damage of the testis, reduced cell apoptosis in testis tissues, and ameliorated the expression of the predicted hub targets (TP53, TNF-α, AKT1, NFκB, and IκBA) and the apoptosis related proteins (Bcl-XL, Bcl-2, Bax, Caspase 3, and Caspase 9). These results indicated that the WZYZP has a protective effect on spermatogenesis disorder, suggesting that it could be an alternative choice for male infertility therapy.

## Introduction

Male infertility is in crisis as a human reproductive health-related issue, accounting for approximately 40% of the infertility cases worldwide (Jonge and Barratt, 2019). Evidence from global reports have also described declining sperm counts and a persisting upward trend in male reproductive system abnormalities (Kilchevsky and Honig, 2012; Barratt et al., 2017). Factors such as genetics, endocrinopathies, lifestyle, and adverse environmental exposure seem to be responsible for the risks of male infertility (Brugh et al., 2003; Skakkebaek et al., 2016). Spermatogenesis is a complex process in which functional sperm is created from initially undifferentiated germ cells by continuous mitosis, meiosis, and cell differentiation in the testis (Zhao W. P. et al., 2018). Abnormal progress in these steps results in disorders of spermatogenesis, and thus male reproductive abnormalities appear. Since male infertility can cause a serious social crisis, many studies have been carried out in this field, and many treatments have also been proposed. Currently, surgical and medical management are still the main strategies for male infertility treatment (Schiff et al., 2007). Compared to surgical therapy, which is mainly focused on overcoming anatomical defects and pathophysiology abnormalities, medical therapy is available to cure hormonal abnormalities, improve sperm quality, and remedy sexual dysfunction (Brannigan, 2019).

Traditional Chinese medicine (TCM) offers experienced and alternative treatment options, and its efficiency in male infertility has been proved in clinical practices by a long history of application (Jiang et al., 2017; Zhou et al., 2019). The Wuzi Yanzong pill (WZYZP) is a classical TCM prescription composed of five traditional Chinese herbs with the efficacy of kidney reinforcement and essence benefit to ameliorate male reproductive dysfunctions, including impotence, asthenospermia, oligospermia, spermatorrhea, and premature ejaculation. Clinical studies have shown that the WZYZP could improve semen qualities in male patients with idiopathic infertility or poor semen parameters, and it also has a significant effect on the treatment of oligospermia by improving several semen parameters and decreasing DNA damage in oligospermia patients (Zhao M. P. et al., 2018; Li et al., 2019; Zhao M. P. et al., 2019). However, the pharmacological mechanisms of the WZYZP on spermatogenesis disorder in male infertility has not been investigated extensively.

**FIGURE 1:**
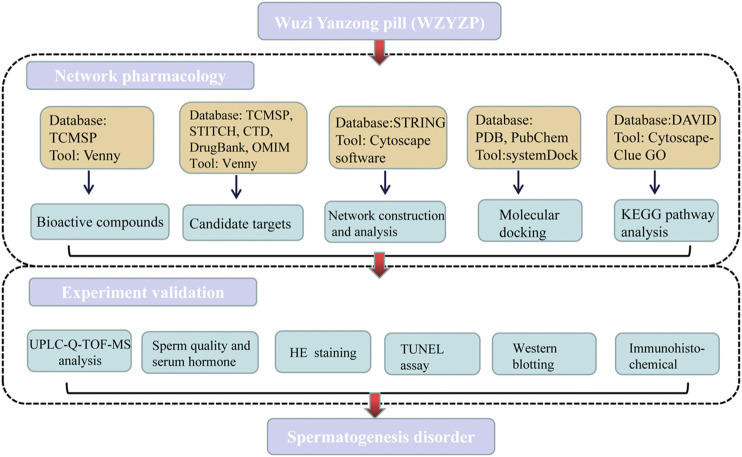
The detailed flow chart of the study design.

With the rapid development of bioinformatics and public biomedical databases, network pharmacology has strongly facilitated the understanding of molecular mechanisms of TCM from a holistic and systemic perspective (Luo et al., 2020). Network pharmacology analysis can provide comprehensive information on chemical, biological, and pharmacology properties, chemically related targets, and disease networks of the TCM. In the present study, we applied network pharmacology to analyze bioactive compounds and targets of the WZYZP, and the underlying mechanisms of the WZYZP in spermatogenesis disorder. Further experiment model was also established to evaluate the effect of WZYZP on spermatogenesis disorder. The flow chart of the current study is shown in Figure 1.

## Materials and Methods

### Screening the Bioactive Compounds of Wuzi Yanzong Pill

The WZYZP is composed of five traditional Chinese herbs, including Chinese wolfberry (*Lycium barbarum L.*, Chinese name Gouqizi), dodder seed (*Cuscuta chinensis Lam.*, Chinese name Tusizi), Schisandra chinensis [*Schisandra chinensis* (*Turcz.*)*Baill.*, Chinese name Wuweizi], raspberry (*Rubus idaeus L.*, Chinese name Fupenzi), and plantaginis semen (*Plantago asiatica L.*, Chinese name Cheqianzi). The detailed chemical information on these herbs was searched in the TCM systems pharmacology database and analysis platform (TCMSP) database (http://ibts.hkbu.edu.hk/LSP/tcmsp.php), including the compound’s chemical name, molecular weight, ADME parameters [oral bioavailability (OB), drug likeness (DL), and intestinal epithelial permeability (Caco-2)], CAS ID, ingredient’s related targets, diseases, etc. According to the suggestion from the TCMSP, an OB ≥30%, DL index ≥0.18, and Caco-2 ≥ −0.4 were chosen as the criteria for screening the bioactive components of the WZYZP (Huang et al., 2014).

### Prediction of Candidate Targets From the Wuzi Yanzong Pill for Spermatogenesis Disorder Treatment

Based on the selected bioactive components in the WZYZP, component related proteins were searched and predicted from the TCMSP database and STITCH database (http://stitch.embl.de/cgi/input.pl?UserId=5db2B3xcD7Am&sessionId=MJAacSCEy4Ac). Spermatogenesis disorder—related proteins were screened from the DrugBank (http://ctdbase.org/), CTD (http://ctdbase.org/), and OMIM (https://www.omim.org/) databases. Then the names of the proteins were further standardized with the Uni Prot KB (http://www.uniprot.org), and the overlapping proteins from component-related and disease-related proteins were identified as the candidate targets of the WZYZP for spermatogenesis disorder treatment.

### Network Construction and Analysis

Based on the selected compounds and their related targets, the herb-compound-target network of the WZYZP was constructed using Cytoscape software. A protein–protein interaction (PPI) network of the target was also constructed. The STRING database (https://string-db.org/) was used to explore the interactions between the proteins, and then the interaction data obtained from STRING was imported into the Cytoscape software to visualize the PPI network. Topological properties of the PPI network were also analyzed to screen hub candidate targets.

### Molecular Docking

The crystal structure of the hub target was obtained from the RCSB Protein Data Bank (http://www.rcsb.org/), and the 2D structure of the test compound was downloaded from the PubChem database (https://pubchem.ncbi.nlm.nih.gov/). The systemDock online tool (http://systemsdock.unit.oist.jp/iddp/home/index) was used to test the docking between hub proteins and related compounds. The molecular docking result was presented as the docking score with a high docking score indicating the tested protein could act as an ideal target of a compound from WZYZP.

### Gene Functional Enrichment Analysis of the Proteins

To further understand the underlying mechanisms of WZYZP as therapeutic agent for spermatogenesis disorder, biological functions of the identified targets were analyzed. Gene ontology (GO) and Kyoto Encyclopedia of Genes and Genomes (KEGG) pathway enrichment analyses of these targets were performed using the DAVID database (https://david.ncifcrf.gov/content.jsp?file=WS.html) and Cytoscape software plugin tool Clue GO. The targets list was inputted and a significance level of *p* < 0.05 was selected. The biological processes and pathways enriched by these candidate targets were presented in the corresponding network.

### Preparation of Wuzi Yanzong Pill

WZYZP prescription is referenced by the Chinese Pharmacopoeia (2015) and composed of five traditional Chinese herbs: Chinese wolfberry (30 g, batch number 180802), dodder seed (30 g, batch number 180301), Schisandra chinensis (10 g, batch number 180401), raspberry (15 g, batch number 180901), and plantaginis semen (10 g, batch number 190501). The prepared pieces of these five herbs were obtained from Zhejiang Huisong Pharmaceuticals Co., Ltd. (Hangzhou, China) in the same batch and further mixed and prepared according to the weight ratio aforementioned to obtain the prescription of the WZYZP by the TCM Dispensary of Zhejiang Provincial Integrated Chinese and Western Medicine Hospital (Hangzhou, China). The final prepared WZYZP was sealed and stored in a dry and cool location. For experiment, the pill was ground to powder and dissolved with saline at concentration of 0.35 and 0.7 g/ml for further rat administration.

### Chemical Characterization Analysis of Wuzi Yanzong Pill Using Liquid Chromatography to Quadrupole/Time-of-Flight Mass Spectrometry

Liquid chromatography to quadrupole/time-of-flight mass spectrometry (UPLC-Q/TOF-MS) analysis for WZYZP was performed on a Waters ACQUITY I-Class Plus UPLC system (Waters, United States) coupled with a SCIEX X-500R Q/TOF-MS system (AB SCIEX, United States). An ACQUITY UPLC BEH C18 column (2.1 mm × 100 mm, 1.7 μm; Waters, United States) was used with a mobile phase comprising 0.1% formic acid-containing acetonitrile (A) and 0.1% aqueous formic acid (B) for the following gradients: 0–2 min, 99% B; 2–12 min, 99–55% B; and 12–17 min, 55–15% B. The flow rate was set to 0.3 ml/min, and the injection volume was 2 μl. The Q/TOF-MS system used an electrospray ionization (ESI) source (AB SCIEX, United States) with negative and positive modes. The specific conditions were set as follows: Ion Source Gas1 (Gas1): 55, Ion Source Gas2 (Gas2): 45, Curtain gas (CUR): 35, source temperature: 600°C, Ion Sapary Voltage Floating (ISVF):5,500 V/−4,500 V; TOF MS scan m/z range: 100–1,500 Da, production scan m/z range: 25–1,500 Da, TOF MS scan accumulation time 0.25 s/spectra, and product ion scan accumulation time 0.035 s/spectra. Secondary mass spectrometry was acquired by information-dependent acquisition (IDA) in the high sensitivity mode and the declustering potential (DP) was ±60 V (positive and negative mode). The obtained mass data was analyzed using SCIEX OS software. SCIEX OS was developed by AB SCIEX with the advantages of easy operation, high efficiency, data accuracy and integrity. This software contains multiple confidence criteria, including quality accuracy, retention time, isotopes, and matches with compound libraries. In this study, based on SCIEX OS software, the compounds in WZYZP could be identified according to the first-order accurate mass number, isotope distribution ratio, and MS/MS data of the compounds and the TCM MS/MS library (including secondary data of more than 1,500 Chinese herbal medicines).

### Animals

Male Sprague-Dawley rats weighing 250 ± 20 g were purchased from the Center of Experimental Animals at the Shanghai Sippr-BK Laboratory Animal Co. Ltd. (Shanghai, China). The animal experiments were approved by the Ethics Committee of Zhejiang Traditional Chinese Medicine University (Hangzhou, China). All animal procedures were in accordance with the National Institutes of Health Guide for the Care and Use of Laboratory Animals. All rats were housed at a standard room temperature of 23 ± 2°C and humidity of 55–70% under a 12 h light/dark cycle with access to food and water *ad libitum*.

### Experiment Design and Drug Administration

After a week of adaptive feeding, SD rats were randomly divided into two groups: sham group and model group. Model group rats were injected with tripterygium glycosides (TGs) at a dose of 40 mg/kg/d (i.g.) for four consecutive weeks (Zhang et al., 2018). A spermatogenesis disorder rat model was confirmed with the characteristics of testicular pathology, semen concentration, and semen motility. SD rats in the sham group (*n* = 12) were injected with same volume of saline for 4 weeks. Subsequently, the model rats were randomly divided into four groups (12 rats in each group): 1) model group: model rats treated with distilled water; 2) WZYZP 0.7: model rats were treated with WZYZP at a dosage of 0.7 g/kg/d; 3) WZYZP 1.4: model rats were treated with WZYZP at a dosage of 1.4 g/kg/d; and 4) clomiphene citrate (CC): model rats were treated with CC at a dosage of 30 mg/kg/d. Additionally, the sham group rats were treated with distilled water and served as a normal control. The rats were daily administrated with WZYZP, CC or distilled water for another 4 weeks after the model was established. After 4 weeks of treatment, all rats were anesthetized with chloral hydrate (350 mg/kg, i.p.), and blood samples were collected from the abdominal aorta. The testes and epididymides were also removed; then the left testis was fixed in 4% paraformaldehyde for histological analysis, and the right testis was frozen in liquid nitrogen for protein extraction.

### Enzyme-Linked Immunosorbent Assay

Concentrations of serum hormone levels, including testosterone (T, E-EL-0155c, Elabscience Biotechnology Co., Ltd., China) and follicle-stimulating hormone (FSH, MM-0566R1, MEIMIAN, China), were detected by Enzyme-Linked Immunosorbent Assay assay using the corresponding commercial kits according to the manufacturer’s protocols.

### Sperm Quality Test

The sperm count and motility were evaluated as previously described (Ji et al., 2016). Briefly, the rat’s epididymis was cut into small pieces, and each 5 μl sperm sample was diluted with 95% diluent. The diluent was made of 5 g sodium biocarbonate, 1 ml of 35% formalin, and 25 mg of eosin per 100 ml diluted water. Approximately 10 μl of diluted sperm suspension was transferred to each counting chamber of the neubauer hemocytometer and was counted under a light microscope (AE2000, Motic) to determine the sperm concentration. For sperm motility, 10 μl of sperm suspension was placed onto a glass slide, and the number of sperm cells was recorded as either mobile or immobile under a light microscope.

### Hematoxylin and Eosin Staining and Terminal Deoxynucleotidyl Transferase dUTP Nick End Labeling Assay

Rat testes were fixed in paraformaldehyde for 48 h and embedded in paraffin. Then the testis tissues were cut into 5 μm thick sections. The sections were stained with hematoxylin and eosin and observed under a light microscope (AE2000, Motic) at ×200 and ×400 magnification. A TUNEL assay was used to detect the apoptosis rate of testicular cells using an apoptosis detection kit, (*In Situ* Cell Death Detection POD Kit; Roche, Germany) according to the manufacturer’s instructions. Briefly, testis tissue sections were treated with 20 μg/ml proteinase K for 15 min at 37°C. They were then rinsed with PBS three times and added to the TUNEL reaction mixture for 60 min at 37°C. After the sections were blocked with 3% BSA solution, 50 μl of secondary antibody were placed onto the sections and incubated at 37°C for 30 min. Next, the sections were washed again and incubated with 100 μl of DAB substrate for 10 min at room temperature. After that, sections were rinsed with PBS and further counterstained with hematoxylin. The TUNEL positive cells were stained brown.

### Western Blotting

Testis tissues were lysed in ice-cold RIPA buffer (Beyotime Institute of Biotechnology, Hangzhou, China) for 30 min, the lysates were centrifuged at 12,000 × g for 10 min, and then the supernatant solution was collected. The protein concentration of the supernatant was detected with a BCA kit (Solarbio). Protein samples were then loaded on 5% SDS-PAGE and transferred to PVDF membranes. The membranes were blocked with 5% nonfat milk for 2 h at room temperature and then washed three times with TBST. Next, the membranes were incubated with the specific primary anti-bodies (Abcam, United States) including anti-TP53 (ab202026, 1:2000), p-AKT1 (ab81283, 1:5000), TNF-α (ab6671, 1:1000), p-NF-κB p65 (ab222494, 1:1000), p-IκBA (ab133462, 1:5000), Bcl-XL (ab32370, 1:1000), Bcl-2 (ab32124, 1:1000), Bax (ab32503, 1:5000), cleaved-Caspase 3 (ab32042, 1:1000), and cleaved-Caspase 9 (ab2324, 1:2000) at 4 C overnight. Afterwards, the membranes were washed with TBST and further incubated with horseradish peroxidase (HRP)-conjugated goat anti-rabbit lgG antibody (ab205718, 1:2000) for 1 h at room temperature. The protein bands were visualized with an enhanced chemiluminescence (ECL) system, and the relative intensity of the band was quantified with Image J software (National Institutes of Health, United States). β-actin was used as the internal control to normalize the relative band density.

### Immunohistochemistry Staining

Deparaffinized and hydrated testicular tissue sections were treated with hydrogen peroxidase for 15 min at room temperature and blocked with 10% normal goat serum. Then the sections were incubated with antibodies against Bcl-XL (ab32370) and Bax (ab32503), overnight at 4 C. After washing, the sections were further incubated with the biotinylated HRP-conjugated goat anti-rabbit Ig G antibody (ab205718) for 10 min at room temperature, the sections were rinsed with PBS and stained with DBA solution for 3–10 min according to observation under a light microscope. After terminating the reaction with distilled water, the sections were further counterstained with hematoxylin for 3 min, the tissue section in each group were observed under a microscope and photographed.

### Statistical Analysis

The experimental data are presented as the mean ± standard deviation (SD). Statistical analysis was performed with SPSS 18.0 software. Statistical significance between groups was calculated by One-way ANOVA followed by hos post LSD test. *p* < 0.05 was considered to be statistically significant.

## Results

### Screening of Potential Bioactive Compounds and Candidate Target in Wuzi Yanzong Pill

**FIGURE 2:**
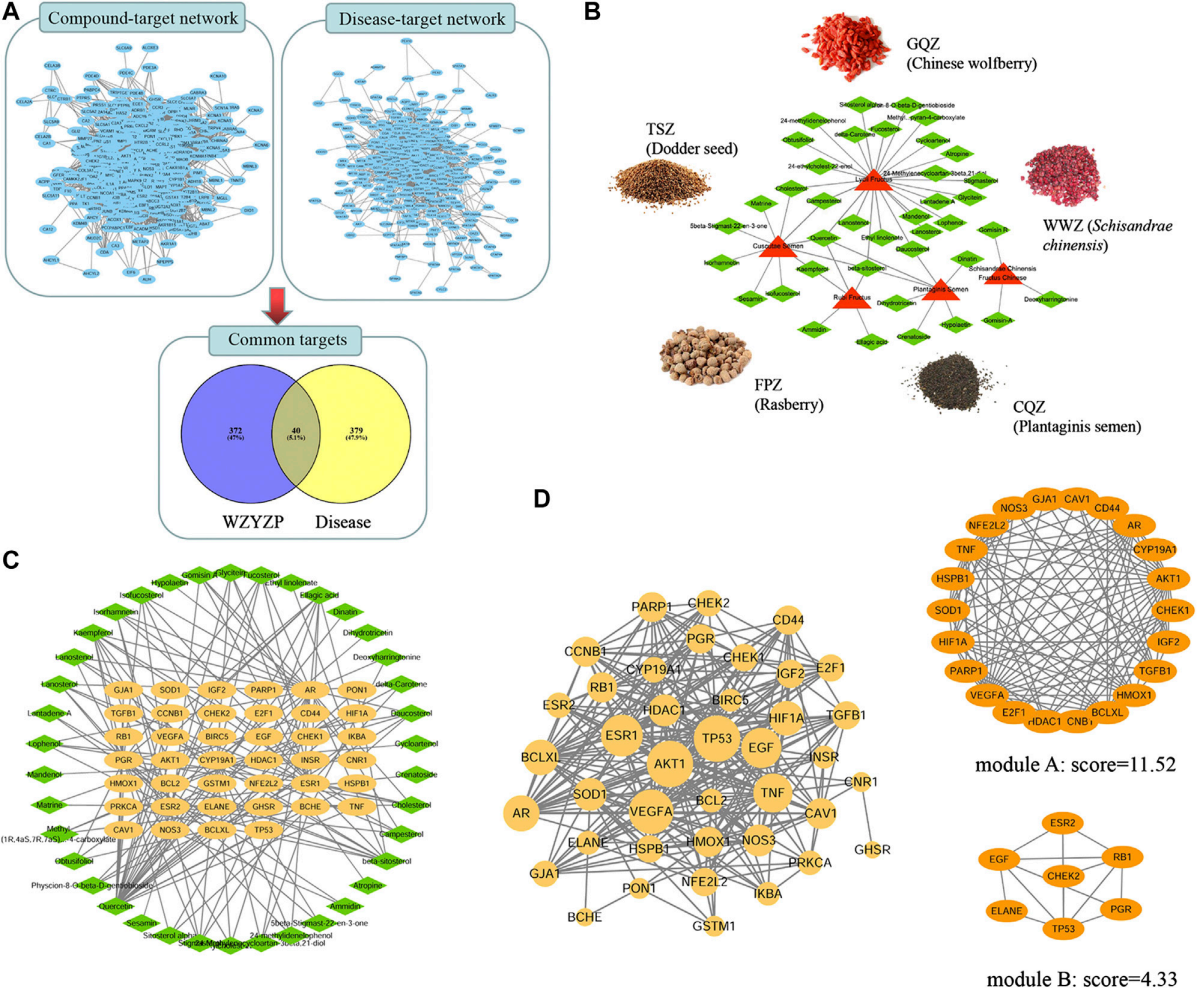
Network Pharmacology analysis of the WZYZP for spermatogenesis disorder treatment. **(A)** Candidate target screening of the WZYZP for spermatogenesis disorder treatment. **(B)** Herb-compound network of the WZYZP. The red triangle represents different herbs in the WZYZP, and green diamond nodes represent active compounds. **(C)** The compound-target network of the WZYZP. The orange node represents the target, and green diamond nodes represent active compounds from different herbs in the WZYZP. **(D)** PPI network of the targets. There are 40 nodes and 288 edges in the PPI network, some targets (AKT1, TP 53, VEGFA, ESR1, EGF, and TNF) were identified with high node degrees. Two modules were screened from the PPI network, and the module scores are 11.52 and 4.33, respectively.

Based on the TCMSP database and the criterion of ADME parameters (OB, DL, Caco-2), a total of 72 bioactive compounds were retrieved from WZYZP. Based on the screened compounds in the WZYZP, 412 related targets were predicted. For the disease “spermatogenesis disorder,” a total number of 419 targets were also screened. Furthermore, a Venny assay showed that 40 targets overlapped between compound related targets and disease related targets. They were thus identified as the candidate WZYZP-related targets for spermatogenesis disorder treatment (Figure 2A). And 39 out of the aforementioned 72 compounds targeted these 40 identified proteins, thus these 39 compounds were selected as the bioactive compounds from the WZYZP for spermatogenesis disorder treatment (Figure 2B). The WZYZP compound-target network was also showed in Figure 2C, with the 39 bioactive compounds targeting 40 candidate proteins included, forming 152 edges in the network. Some compounds targeted several proteins simultaneously, and some proteins were also targeted by more than one compound, thus forming a tight compound-target network.

### Protein–Protein Interaction Network Analysis

The interactions between targets were analyzed with the constructed PPI network (Figure 2D). 40 nodes and 288 edges comprised this network, with an average node degree of 7.2. The node degree was positively represented by node size. Notably, the network comprised some high-degree targets, including TP53 (also called p53, degree = 32), AKT1 (degree = 32), VEGFA (degree = 30), ESR1 (degree = 27), EGF (degree = 25), TNF (degree = 24), AR (degree = 22), and Bcl-XL (degree = 21). Two significant modules were screened from the network: module A comprised 22 targets and 121 edges, five out of these targets were the formerly mentioned high-degree targets, including, AKT1, VEGFA, TNF, AR, and Bcl-XL; module B comprised seven targets and 13 edges, and two out of these targets were the formerly mentioned high-degree targets, including TP53 and EGF.

### Predicted Binding of the Bioactive Compounds to the Hub Targets

**FIGURE 3:**
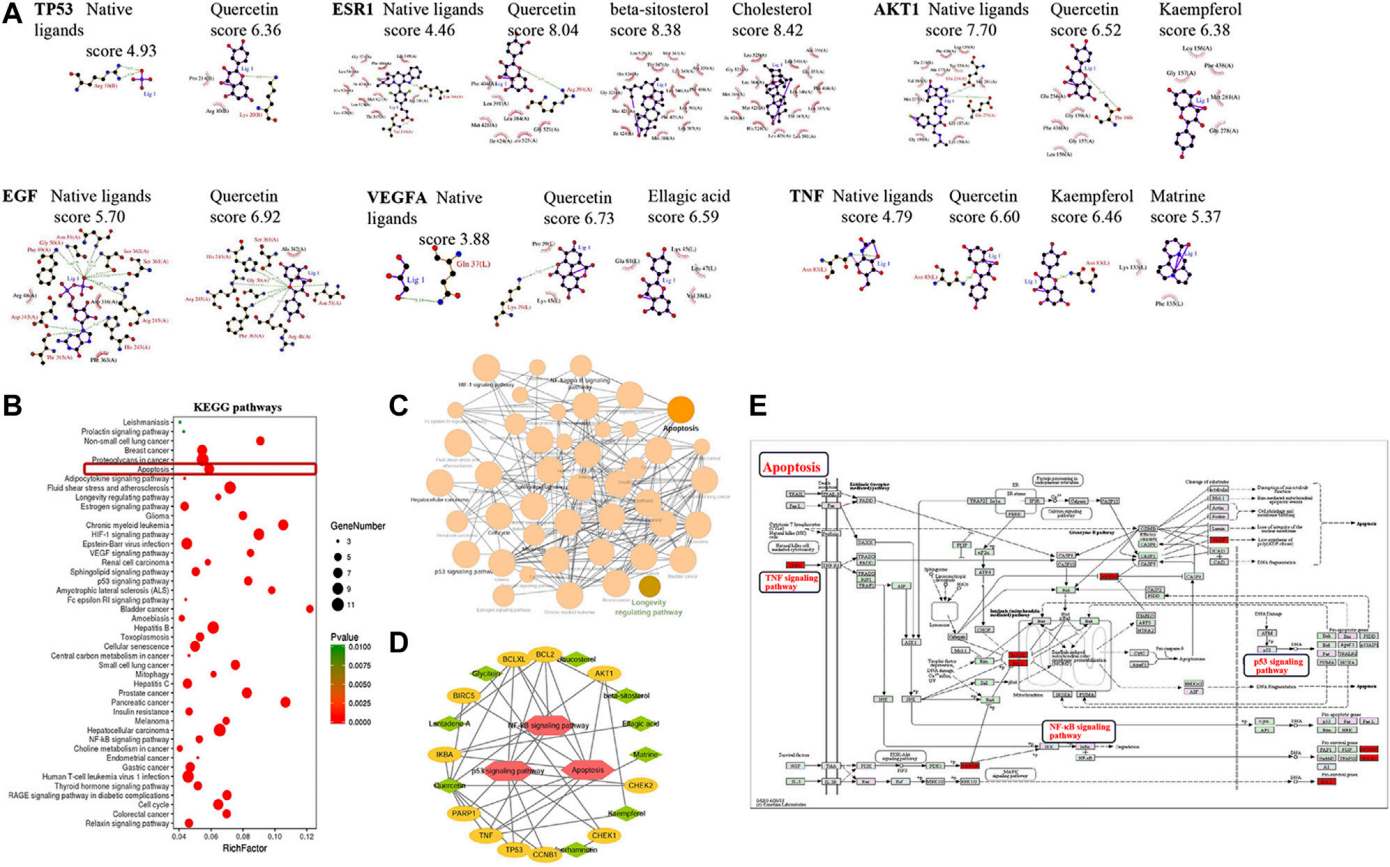
Molecular docking and KEGG pathway analysis of the targets. **(A)** Molecular docking of the hub targets and related bioactive components. The interactive hub targets (TP 53, AKT1, VEGFA, ESR1, EGF, and TNF) and their related ingredients or native ligands were analyzed using molecular docking, a higher docking score indicates stronger binding. **(B)** Bubble chart of the targets enriched KEGG pathways. The bubble size represents the number of the target in the enriched pathway terms, bubble color represents the pathway’s *p* value. The apoptosis pathway was founded to have a high number of targets enriched and a significant *p* value. **(C)** KEGG pathway network of the targets. Different nodes represent different pathways, these pathways interact with each other and form a pathway network. The apoptosis pathway interacts with multiple pathways. **(D)** Pathway- target-ingredient network. The red polygon represents the pathway, the orange node represents the target, and the green diamond nodes represent active ingredients in WZYZP. **(E)** The apoptosis and its associated proteins or singling pathways. Data were retrieved from the KEGG database (https://www.genome.jp/kegg/).

Molecular docking tests were performed to evaluate the binding between the associated bioactive compounds and the high-degree targets TP53, AKT1, VEGFA, ESR1, EGF, and TNF. The binding results are presented by docking score (Figure 3A). Most of the compounds targeted with tested proteins that have higher binding scores than native ligands. For TP53, the binding score was 6.36 with quercetin, and for the native ligand, it was 4.93; for TNF, the binding score was 6.60 with quercetin, 6.46 with qaempferol, 5.37 with matrine, and 4.93 with the native ligand.

### Target Enriched Kyoto Encyclopedia of Genes and Genomes Pathway Analysis

Totally, 44 pathways were found to be significantly associated with the input set of 40 targets. From the bubble chart of the target enriched pathways, most of these pathways were enriched by multiple targets (Figure 3B). The apoptosis pathway was enriched by eight targets: TP53, TNF, Bcl-2, Bcl-XL, IκBA, BIRC5, and PARP1 with a *p* value <0.001. Pathway network showed that these pathways interacted closely with each other, and the apoptosis pathway interacted with multiple pathways, including the NF-κB pathway and p53 signaling pathway (Figure 3C). The apoptosis pathway was therefore selected for further analysis. The pathway-target-compound network showed that the apoptosis and it associated pathways (such as NF-κB, p53 signaling pathways) were enriched by multiple targets, including TP53, TNF, AKT1, Bcl-XL, BCL-2, and IκBA (Figure 3D). These associated targets bound to related compounds, including quercetin, beta-sitosterol, daucosterol, and kaempferol, forming a tight network. The apoptosis pathway and its related proteins or signaling pathways are presented in Figure 3E, which were retrieved from the KEGG database (https://www.genome.jp/kegg/). It could be observed that some proteins, such as TNF-α, p53, NF-κB, BCL-2, Bax, Bcl-XL, caspase 3, and caspase 9, and pathways, such as the NF-κB, p53, TNF, and PI3K/AKT signaling pathways, were involved in the apoptosis pathway.

### Chemical Characterization of Wuzi Yanzong Pill

**FIGURE 4:**
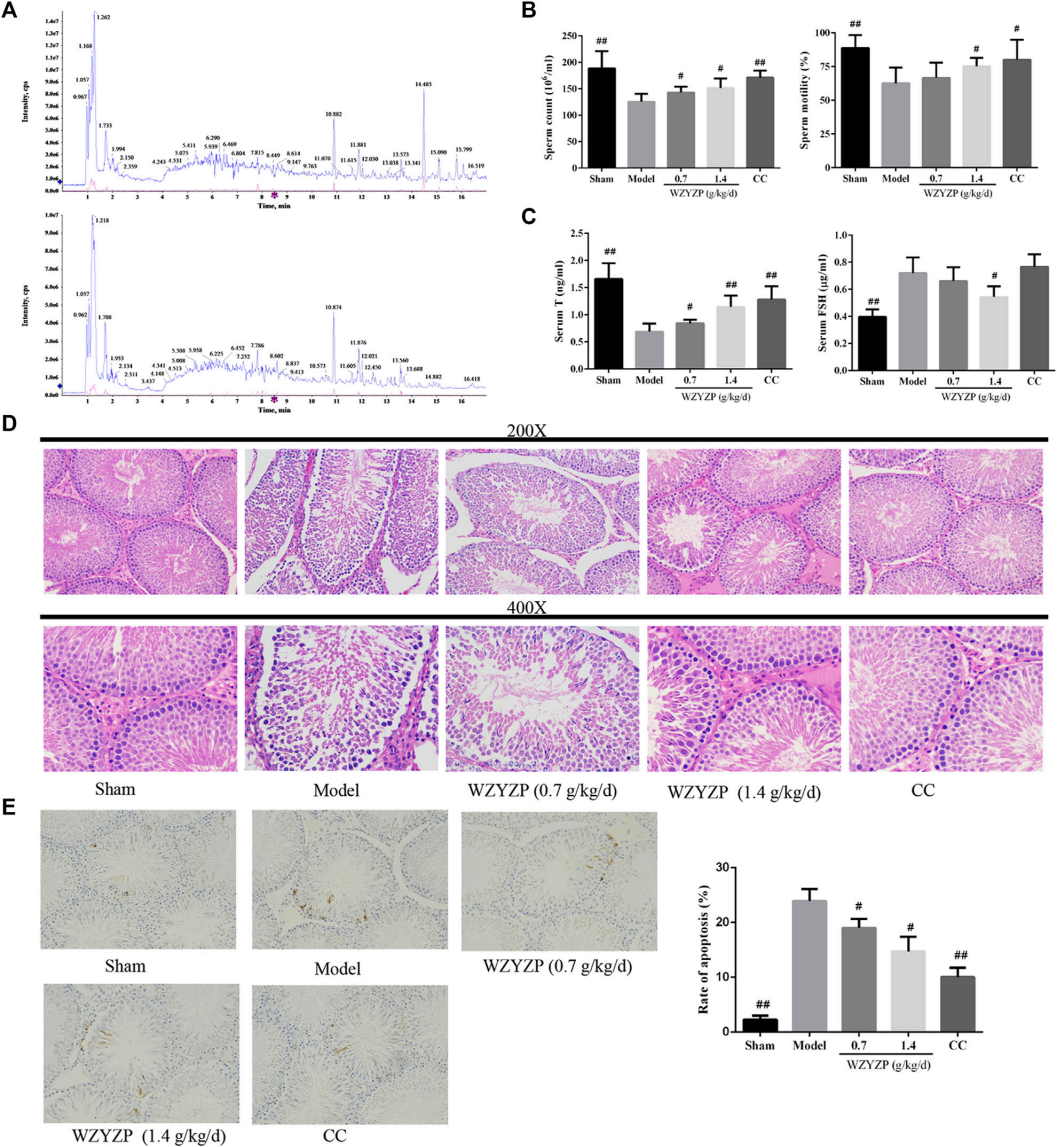
Compounds identification and the effect of the WZYZP on the experimental spermatogenesis disorder model rats. **(A)** The phytochemical compositions identification in the WZYZP by UHPLC-Q-TOF/MS in the positive ion mode and negative ion mode. **(B)** Effect of the WZYZP on sperm counts and motility. **(C)** Effect of WZYZP on serum hormone levels of T, FSH levels were detected with an ELISA assay. **(D)** HE staining to evaluate the effect of the WZYZP on rat testes histological changes. Above, magnification ×200, 400. **(E)** TUNEL staining for the evaluation of cell apoptosis. Above, magnification ×200. ^#^p < .05 vs. Model group, ^##^p < .01 vs. Model group.

The chemical characterization of WZYZP was analyzed with UPLC-Q/TOF-MS and the results were presented in Figure 4A and Supplementary Material S1. The obtained mass data was analyzed using SCIEX OS software. According to the first-order accurate mass number, isotope distribution ratio and MS/MS data of the compounds and the TCM MS/MS library in SCIEX OS software, 40 compounds were identified under positive ion mode, and 41 compounds were identified under the negative ion mode from WZYZP. Some of compounds were identified both under positive ion mode and negative ion mode. These compounds mainly belonged to flavonoids, alkaloids, glycosides, acids, phenols, etc. In addition, some of the compounds identified by UPLC-Q/TOF-MS were coincident with the aforementioned compounds screened from the TCMSP database in WZYZP. Such as betaine, nicotinic acid, quercetin, schisanhenol, schisandrin, tiliroside, rutin, succinic acid, protocatechuic acid, esculetin, citric acid, ellagic acid, geniposidic acid, hyperin, etc. Mass spectrum of the identified compounds were also provided in Supplementary Material S2.

### Wuzi Yanzong Pill Treatment Improved Sperm Quality and Attenuated Serum Hormone Levels

Based on the analysis of network pharmacology, an experimental model was established to further verify the effect and potential mechanisms of WZYZP in spermatogenesis disorder. The results showed that the 4-week TGs administration reduced sperm concentrations and motility significantly in the model group rats compared to the sham group (*p* < 0.01; Figure 4B), indicating that the spermatogenesis disorder model was successfully established. After WZYZP treatment, the results showed that the sperm counts were significantly increased in the WZYZP 0.7 g/kg and WZYZP 1.4 g/kg groups (*p* < 0.05, *p* < 0.01, respectively), and the sperm motility was significantly increased in the WZYZP 1.4 g/kg group (*p* < 0.05). As for serum hormone levels (Figure 4C), T levels were significantly increased in the WZYZP 0.7 g/kg and WZYZP 1.4 g/kg groups (*p* < 0.05, *p* < 0.01, respectively).

### Wuzi Yanzong Pill Treatment Mitigated the Histopathological Damages and Cell Apoptosis of the Testis Tissues

In histological analysis (Figure 4D), the testicular tissue of the sham group revealed a normal arrangement of the spermatogenic cells in the seminiferous tubules, without any histopathological lesions. In contrast, the model group exhibited significant testicular tissues histopathological damage, including exfoliation, intertubularoedema karyorrhexis of the seminiferous tubules, unordered arrangement of spermatogenic cells, and vacuolization in the cytoplasm of sertoli cells. WZYZP treatment (0.7 and 1.4 g/kg groups) partly attenuated the testicular tissue histopathological damages, with the most improvement in the high dose group. A TUNEL assay was also performed to assess the effect of the WZYZP on cell apoptosis in testis tissues, with the TUNEL-positive brown stain as cell representative. As Figure 4E revealed, the percentage of TUNEL-positive cells in the model group was significantly higher than those in the sham group (*p* < 0.01), and WZYZP treatment significantly reduced the increased percentage of apoptotic cells compared with the model group (*p* < 0.05).

### Wuzi Yanzong Pill Treatment Reduced the Expression of the Predicted Hub Targets

**FIGURE 5:**
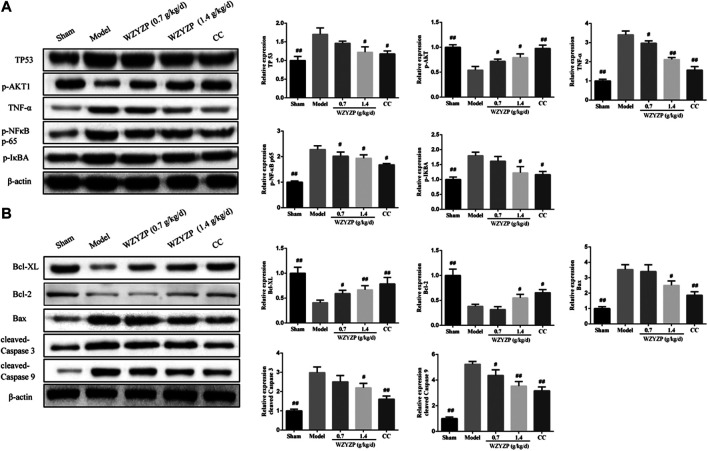
Effect of the WZYZP on the hub targets and apoptosis pathway. **(A)** Protein expression of TP53, p-AKT1, TNF-α, p-NF-κB p65, and p-IκBA in rat testes. **(B)** Protein expression of Bcl-XL, Bcl-2, Bax, cleaved- Caspase 3, and cleaved- Caspase 9 in rat testes. The protein expression was detected with a western blot assay. β-actin was used as control. ^#^ *p* < 0.05 vs Model group, ^##^ *p* < 0.01 vs Model group.

The expression of TP53, AKT1, TNF-α, NF-κB, and IκBA were further examined, as, in the network pharmacology analysis, these targets showed high-degree nodes in the PPI network and were actively involved in the apoptosis pathway, suggesting that they might play a critical role in the potential mechanism of the WZYZP for spermatogenesis disorder treatment. As the western blotting results indicated (Figure 5A), the protein expression of TP53, TNF-α, p-NF-κB p65, and IκBA in the model group were significantly increased compared to the sham group (*p* < 0.01). And after WZYZP treatment, the proteins expression levels of these targets were significantly reduced (*p* < 0.05). In contrast, the protein expression of p-AKT1 was significantly inhibited in the model group compared to the sham group (*p* < 0.01), and the WZYZP treatment significantly increased the expression levels of these two proteins compared to their levels in the model group (*p* < 0.05).

### Wuzi Yanzong Pill Treatment Ameliorated the Expression of Apoptosis-Related Genes

**FIGURE 6:**
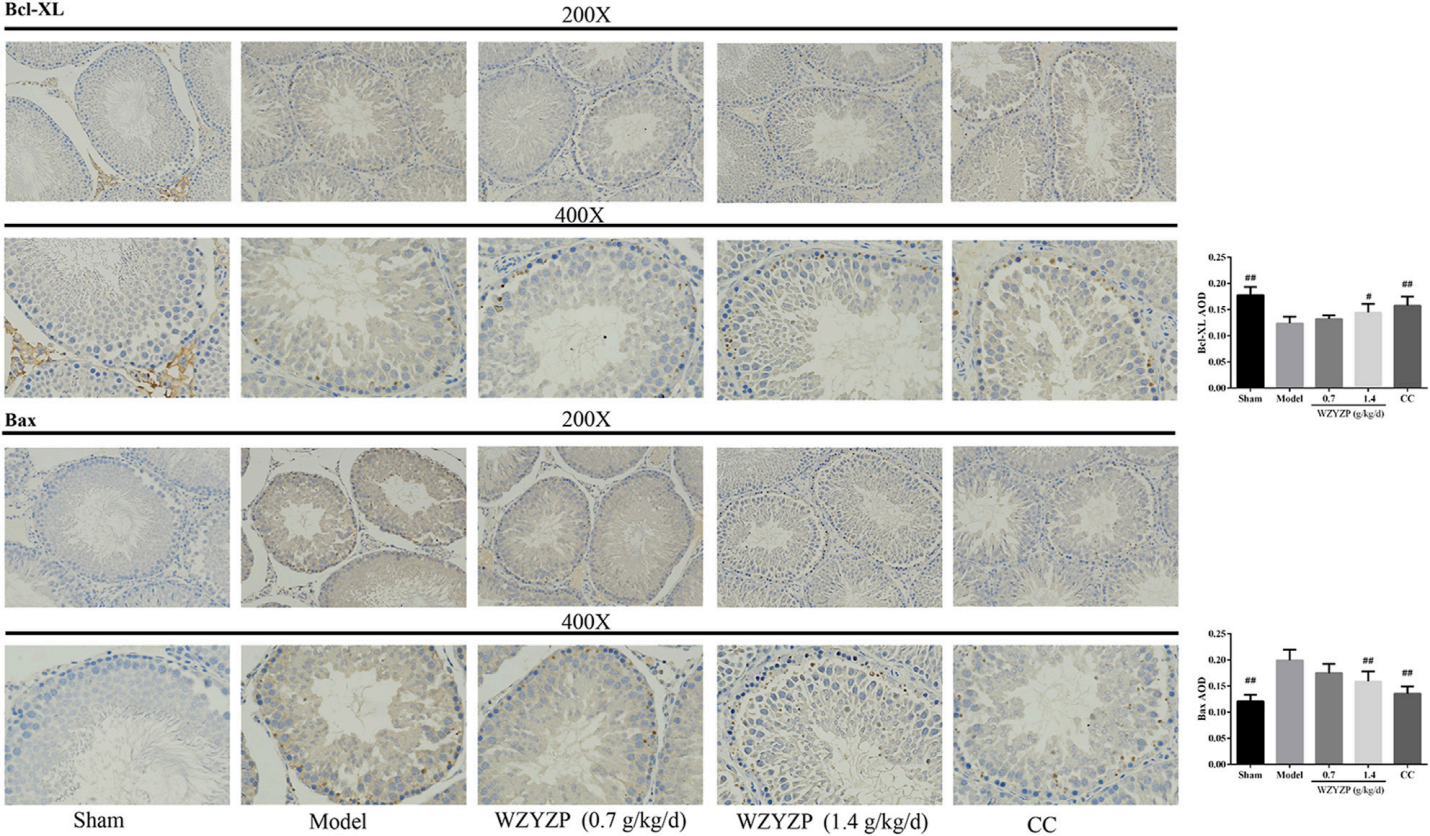
Effect of the WZYZP on the expression levels of Bcl-XL and Bax in testes tissues. The expression levels of Bcl-XL and Bax in the testes were determined by immunohistochemistry analysis. Above, magnification ×200, 400. ^#^p < .05 vs. Model group, ^##^p < .01 vs. Model group.

Due to the network pharmacology analysis, which indicated that the WZYZP attenuates spermatogenesis disorder potentially through the apoptosis pathway, apoptosis-related gene (Bcl-XL, Bcl-2, Bax, Caspase 3, and Caspase 9) protein expression levels were detected in the experimental model. As Figure 5B indicated, the protein expressions of anti-apoptotic genes Bcl-XL and Bcl-2 were significantly inhibited in the model group compared with the sham group (*p* < 0.01), and WZYZP treatment significantly increased gene expression levels in low to high doses compared to the model group (*p* < 0.05). For pro-apoptotic genes, the results showed that the protein expression levels of Bax, cleaved-Caspase 3, and cleaved-Caspase 9 were significantly increased in the model group compared to the sham group (*p* < 0.01), WZYZP treatment significantly inhibited the protein expressions of Bax and cleaved-Caspase 3 in the WZYZP 1.4 mg/kg group, cleaved-Caspase 9 in the WZYZP 0.7 and 1.4 mg/kg group compared to the model group (*p* < 0.05). Immunohistochemistry analysis also showed a similar effect of the WZYZP on the expressions of Bcl-XL and Bax in rat testicular tissues (Figure 6).

## Discussion

Sperm is produced in the testis seminiferous tubules, which are mainly composed of germ cells and sertoli cells. The spermatogenesis is a complex process, with three phases: first, the primitive germ cells or spermatogonia undergo a series of mitotic divisions into spermatocytes; second, the spermatocytes undergo two consecutive divisions in meiosis to produce haploid spermatids; and third, the spermatids are differentiated into functional sperms (Chocu et al., 2012). Many factors could cause testis damage and affect spermatogenesis, including high temperature, radiation exposure, or chemicals, such as cyclophosphamide and TGs. Since the use of TGs showed that it has toxic effects to kidneys and the reproductive system, TGs were thus introduced for experimental spermatogenesis disorder modeling (Du et al., 2018). Experimental studies also find that TGs treatment in male and female rats can cause pathological damages to the testis, epididymis, ovary, and uterus, and TGs can induce sperm abnormality and hormone expression abnormalities (Jing et al., 2017; Guo et al., 2019). In this study, TGs administration in rats for 4 weeks showed that testis tissues exhibited histopathological damage and cell apoptosis. Sperm quality and hormone levels were also affected significantly, which were consistent with the previous studies.

Spermatogenesis disorder is a common but complex disease. To date, many pharmaceutical drugs have been developed for this disorder treatment. TCM therapy has been playing an important role in clinical treatment for a long time (Jiang et al., 2017; Zhou et al., 2019). In this study, network pharmacology analysis was performed to explore the potential bioactive compounds and mechanisms of the WZYZP for spermatogenesis disorders. The results focuses on three aspects: 39 bioactive compounds were identified from the WZYZP; 40 targets of the WZYZP were predicted for spermatogenesis disorder treatment, with tight bindings between the tested targets and their associated compounds; and the KEGG pathway network and pathway-target-compound network revealed that the apoptosis pathway was significantly enriched by multiple signaling pathways, including the p53, TNF, and NF-κB pathways, and multiple targets, including the hub targets TP53, TNF, AKT1, Bcl-XL, Bcl-2, and IκBA.

Within the bioactive compounds screened from WZYZP, flavonoid kaempferol has been reported to attenuate the negative effects from metals on sperm motility (Jamalan et al., 2016). Quercetin has been reported to significantly improve the sperm motility in leukocytospermic patients (Diao et al., 2019). Studies have also demonstrated the protective effect of quercetin on the reproductive system, and *in vivo* administration of quercetin could help to preserve sperm morphology and testicular functions (Nna et al., 2017; Yelumalai et al., 2019). Other bioactive compounds, such as ellagic acid, can also attenuate testicular disruption and sperm damage, which might be associated with targeting inflammatory signals, oxidative stress, and apoptosis (Türk et al., 2010; Arab et al., 2019). Guan et al. study showed that Cuscutae Semen and Lycii Fructus out of WZYZP could improve testicular cell proliferation and inhibits apoptosis in rats with spermatogenic dysfunction (Guan et al., 2020). Except for the studies focused on compounds or herbs in WZYZP, some *in vivo* studies have observed the protective effect of the WZYZP on testicular dysfunction and sperm quality (Ji et al., 2016; Zhao H. et al., 2019). Further research on spermatogenesis disorder experimental model in this study also showed that the WZYZP could attenuate the spermatogenesis disorder in model rats. And the treatment could improve sperm quality, attenuate serum hormone levels, reverse the histopathological damage of the testis, and reduce cell apoptosis in testis tissues significantly. These results suggested that the WZYZP has a potent effect on spermatogenesis disorder, by attenuating testicular and sperm damage, and the bioactive compounds screened from the WZYZP might have a strong contribution to this efficacy.

Network pharmacology and experimental analysis identified several proteins, including TP53 (also called p53), AKT1, TNF-α, and NF-κB, that have important role in WZYZP’s therapeutic effect for spermatogenesis disorder. TP53 and its signaling pathway are functionally important in male and female germ cell survival, both of which affect human infertility (Corbo et al., 2012; Kang and Rosenwaks., 2018). A case-controlled study revealed that the TP53 Ex2+19C>T polymorphism is associated with male infertility in the Chinese population (Huang et al., 2012). Another study showed that male p53−/− Ink4c−/− mice have profoundly reduced fertility, with a marked decreased in sperm count, abnormal sperm morphology and motility, and accumulation of DNA damage (Zalzali et al., 2018). For AKT1, a published study found that the seminiferous tubule diameter in Akt1−/− testes was smaller than that in wild-type mice, and apoptotic sperm were more prevalent in null mice compared to the wild-type mice, whereas the sperm concentration and motility were significantly lower in the null sperm (Kim et al., 2012). The PI3K/AKT signaling pathway also plays an important role in spermatogenesis, targeting this pathway could regulate oxidative stress, apoptosis, sperm motility, as well as Foxo1 nuclear retention in the testis (Sinha et al., 2018; Huang et al., 2019). Inflammation is a significantly factor in testis injury, TNF-α and NF-κB serve as inducers that contribute to spermatogenesis disorders. In mice testis exposed to cadmium, the level of the pro-inflammatory cytokine, TNF-α, in the testis was upregulated, and the alterations in sperm parameters were observed (Li et al., 2016). Many agents can target the NF-κB signaling pathway to prevent the progression of testicular injury by downregulating oxidative stress and inhibiting inflammation (Wang et al., 2018). Network pharmacology in this study identified the bioactive compounds of WZYZP target these proteins in spermatogenesis disorder treatment, suggesting that WZYZP’s therapeutic effect might work through targeting these proteins to regulate testis inflammation, oxidative stress, and apoptosis. Furthermore, it attenuates testis injury and protects sperm quality.

In the pathogenesis of male infertility, evidence-based studies have revealed that there is a critical association with the apoptosis pathway. Apoptosis is an essential physiological process that also occurs in the testis, but the increasing condition of cell apoptosis prevents the normal process of spermatogenesis, thus resulting in a decline of the sperm concentration and motility, and this affects the normal function of the testis (Kim et al., 2002; Shukla et al., 2012). Some signaling pathways, such as the p53 and NF-κB pathways, have been reported to be involved in germ cell apoptosis, and the deactivation of these signaling pathways can effectively rejuvenate testicular function via attenuating germ cell DNA damage and apoptosis (Zhao et al., 2017). In this study, network pharmacology showed that WZYZP-associated targets were critically enriched in the apoptosis pathway, several signaling pathways such as the p53, TNF, and NF-κB pathways were also involved. An *in vivo* experiment showed that the apoptosis related proteins Bax, Caspase 3, and Caspase 9 expression levels were significantly upregulated in the model rats, and anti-apoptosis related proteins Bcl-2 and Bcl-XL expression levels were significantly downregulated in the model rats. There were also histopathological damages and TUNEL positive cells in the testis tissues, suggesting the existence of cell apoptosis in the testis. The cell apoptosis and apoptotic associated protein expression levels were also alleviated with WZYZP treatment. These results indicated that the protective effect of WZYZP on spermatogenesis disorder might have partly contributed to the inhibition of apoptosis by regulating apoptosis associated proteins and signaling. A study investigating the protective effects and underlying mechanism of WZYZP on testicular dysfunction in ageing rats showed that WZYZP effectively downregulated the expression levels of pro-apoptotic proteins p-JNK, Caspase12, and CHOP in testicular germ cell and significantly decreased the numbers of TUNEL-positive cells, which improved age-related testicular dysfunction (Zhao M. P. et al., 2019). In another study, Hu et al. (2019) also demonstrated the protective effects of modified WZYZP on radiated rat sperm were partly due to its ability to intervene in Tip60-p53 mediated apoptosis.

**FIGURE 7:**
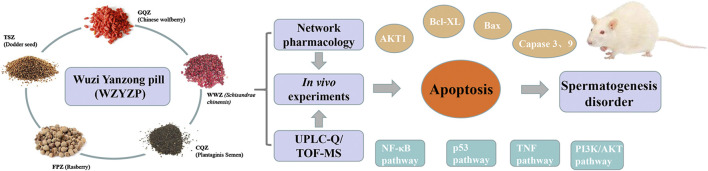
The WZYZP has a curative effect on spermatogenesis disorder via the regulation of apoptosis pathway.

In conclusion, in this study, network pharmacology identified 39 bioactive compounds and 40 related targets for spermatogenesis disorder in WZYZP, forming a tight compound-target network. Further analysis identified TP53, TNF, AKT1, Bcl-XL, BCL-2, and IκBA as hub targets and revealed the apoptosis pathway was significantly involved and enriched by multiple signaling pathways and multiple targets. Additionally, an *in vivo* experiment showed that WZYZP treatment has a protective effect on spermatogenesis disorder by improving sperm quality and reversing histopathological damages of the testis, reducing cell apoptosis in testis tissues, and ameliorating the expressions of the predicted hub targets (TP53, TNF, AKT1, and IκBA) and the apoptosis-related proteins (Bcl-XL, Bcl-2, Bax, Caspase 3, and Caspase 9). Based on network pharmacology analysis and experimental model, this study showed that WZYZP has a curative effect on spermatogenesis disorders, suggesting that it might be an alternative choice for male infertility therapy (Figure 7). However, further in-depth study is still needed to clarify the clear role of WZYZP and identify the complicated mechanism of the WZYZP in male infertility.

## Data Availability

The original contributions presented in the study are included in the article/**Supplementary Material**.
