# Corrigendum: Wuzi Yanzong pill—Based on network pharmacology and *In Vivo* evidence—Protects against spermatogenesis disorder *via* the regulation of the apoptosis pathway

**DOI:** 10.3389/fphar.2022.1129448

**Published:** 2023-01-12

**Authors:** Wang-qiang Chen, Cai-fei Ding, Jia Yu, Chen-ye Wang, Ling-yi Wan, Hui-min Hu, Jian-xiong Ma

**Affiliations:** ^1^ Department of Reproductive Medicine, Zhejiang Provincial Integrated Chinese and Western Medicine Hospital, Hangzhou, China; ^2^ The Second Clinical Medical College, Zhejiang Chinese Medical University, Hangzhou, China; ^3^ Department of Andrology, Dongzhimen Hospital, Beijing University of Chinese Medicine, Beijing, China

**Keywords:** Wuzi Yanzong pill, spermatogenesis disorder, network pharmacology, bioactive compounds, hub targets, apoptosis pathway

In the published article, there was an error in Figures 4, 6 as published. The images in Figure 4D and the immunohistochemical staining of Bcl-XL in Figure 6 were mistakenly swapped. The corrected Figures 4, 6 appear below.

**FIGURE 4:**
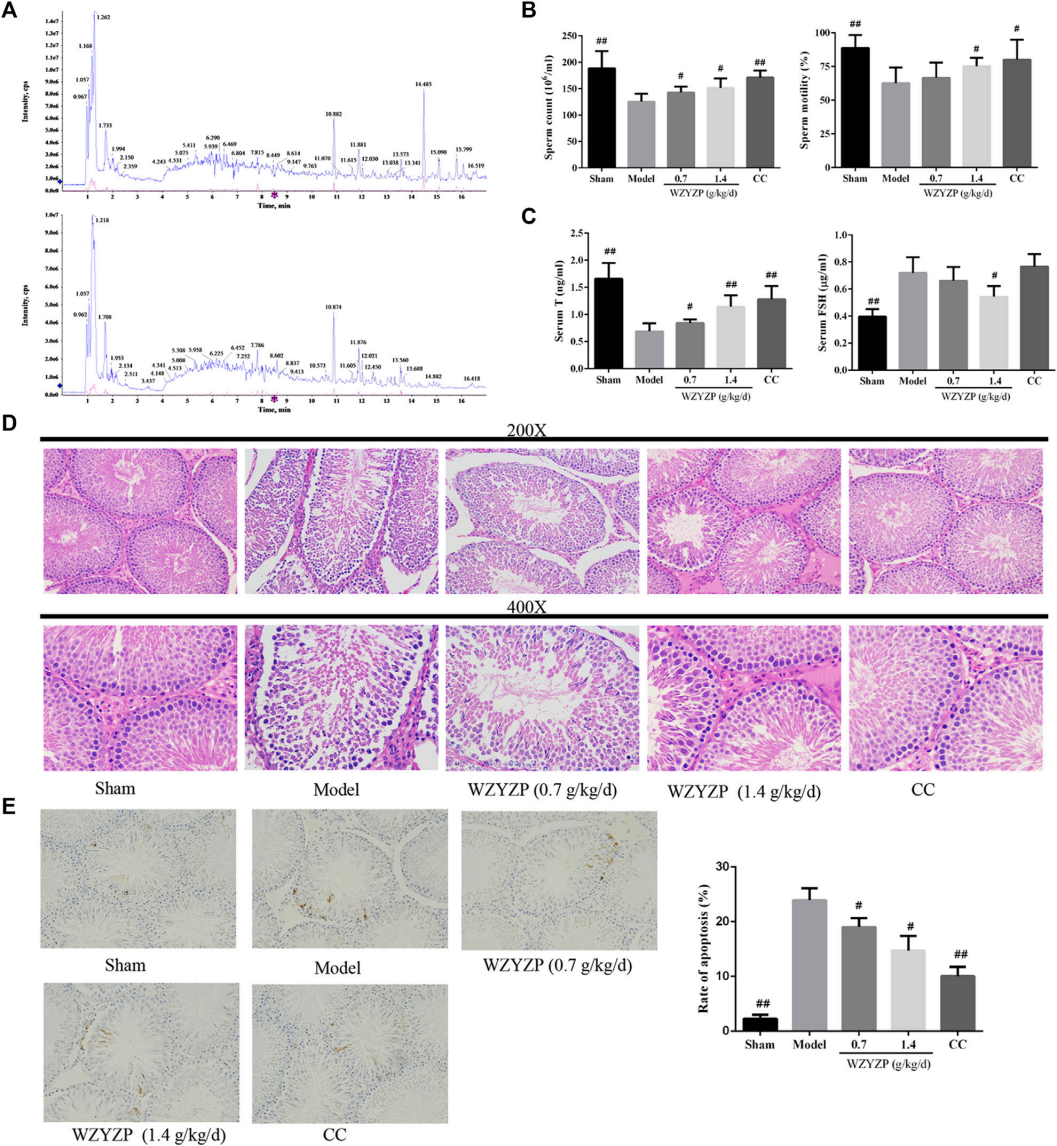
Compounds identification and the effect of the WZYZP on the experimental spermatogenesis disorder model rats. **(A)** The phytochemical compositions identification in the WZYZP by UHPLC-Q-TOF/MS in the positive ion mode and negative ion mode. **(B)** Effect of the WZYZP on sperm counts and motility. **(C)** Effect of WZYZP on serum hormone levels of T, FSH levels were detected with an ELISA assay. **(D)** HE staining to evaluate the effect of the WZYZP on rat testes histological changes. Above, magnification ×200, 400. **(E)** TUNEL staining for the evaluation of cell apoptosis. Above, magnification ×200. #*p* < .05 vs. Model group, ##*p* < .01 vs. Model group.

**FIGURE 6:**
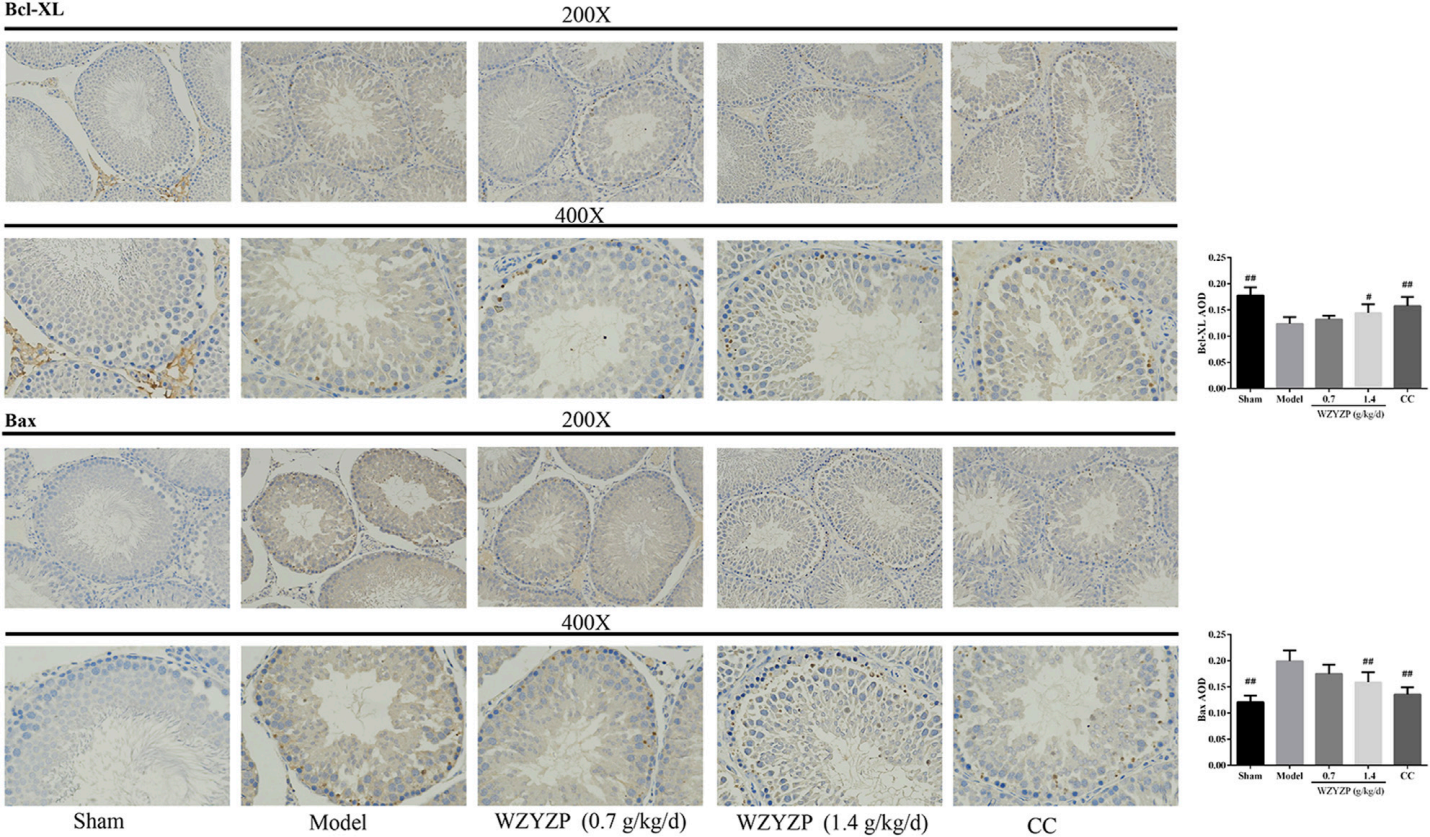
Effect of the WZYZP on the expression levels of Bcl-XL and Bax in testes tissues. The expression levels of Bcl-XL and Bax in the testes were determined by immunohistochemistry analysis. Above, magnification ×200, 400. #*p* < .05 vs. Model group, ##*p* < .01 vs. Model group.

The authors apologize for this error and state that this does not change the scientific conclusions of the article in any way. The original article has been updated.

