# Supplementary material for: Wuzi Yanzong Pill—Based on Network Pharmacology and In Vivo Evidence—Protects Against Spermatogenesis Disorder via the Regulation of the Apoptosis Pathway
Source: Front Pharmacol. 2020 Dec 18;11:592827. doi: 10.3389/fphar.2020.592827 (PMC7775606; doi:10.3389/fphar.2020.592827)
Supplement: Supplementary file 1 [file DataSheet1.doc]

**Supplementary material 1:** Mass spectrometry data and elemental composition of compounds WZYZP by UPLC-Q/TOF-MS analysis

| No. | Retention time (min) | Compounds | Formula | Precursor Mass | Found At Mass | Mass deviation (ppm) | Library Score |
| --- | --- | --- | --- | --- | --- | --- | --- |
| Positive ion mode | | | | | | | |
| 1 | 1.09 | L(+)-Arginine | C_6_H_14_N_4_O_2_ | 175.119 | 175.1193 | 1.7 | 92.1 |
| 2 | 1.17 | Betaine | C_5_H_11_NO_2_ | 118.086 | 118.0863 | 0.2 | 98.6 |
| 3 | 1.21 | Trigonelline | C_7_H_7_NO_2_ | 138.055 | 138.0551 | 0.8 | 94.0 |
| 4 | 1.23 | Proline | C_5_H_9_NO_2_ | 116.071 | 116.0707 | 1.2 | 97.6 |
| 5 | 1.26 | Stachydrine | C_7_H_13_NO_2_ | 144.102 | 144.1022 | 1.9 | 96.6 |
| 6 | 1.27 | Adenine | C_5_H_5_N_5_ | 136.062 | 136.0619 | 0.7 | 81.3 |
| 7 | 1.73 | Nicotinic acid | C_6_H_5_NO_2_ | 124.039 | 124.0395 | 1.7 | 97.9 |
| 8 | 1.73 | Vitamin C | C_6_H_8_O_6_ | 177.039 | 177.0397 | 2.1 | 89.6 |
| 9 | 1.9 | 6-Hydroxypurine | C_5_H_4_N_4_O | 137.046 | 137.0460 | 1.5 | 91.8 |
| 10 | 4.83 | Phenylalanine | C_9_H_11_NO_2_ | 166.086 | 166.0866 | 1.9 | 99.8 |
| 11 | 6.4 | Sesamol | C_7_H_6_O_3_ | 139.039 | 139.0390 | 0.1 | 80.8 |
| 12 | 6.44 | Chlorogenic acid | C_16_H_18_O_9_ | 355.102 | 355.1028 | 1.2 | 100.0 |
| 13 | 7.09 | Vitamin B2 | C_17_H_20_N_4_O_6_ | 377.146 | 377.1461 | 1.5 | 98.0 |
| 14 | 7.52 | Puerarin | C_21_H_20_O_9_ | 417.118 | 417.1196 | 3.8 | 99.2 |
| 15 | 7.98 | Rutin | C_27_H30O_16_ | 611.161 | 611.1620 | 2.2 | 99.6 |
| 16 | 8.18 | Isoquercitrin | C_21_H_20_O_12_ | 465.103 | 465.1035 | 1.5 | 99.6 |
| 17 | 8.18 | Quercetin | C_15_H_10O7_ | 303.053 | 303.0503 | 1.4 | 93.8 |
| 18 | 8.18 | Hyperin | C_21_H_20_O_12_ | 465.103 | 465.1035 | 1.5 | 100 |
| 19 | 8.28 | β-Ecdysone | C_27_H_44_O_7_ | 481.316 | 481.3168 | 1.7 | 93.6 |
| 20 | 8.4 | Isoscopoletin | C_10_H_8_O_4_ | 193.050 | 193.0498 | 1.3 | 94.3 |
| 21 | 8.49 | Aempferol-3-O-rutinoside | C_27_H_30_O_15_ | 595.166 | 595.1669 | 1.9 | 97.6 |
| 22 | 8.57 | Nodakenin | C20H24O_9_ | 409.149 | 409.1500 | 1.7 | 93.5 |
| 23 | 8.63 | Acteoside +NH3 | C_29_H_36_O_15_NH_3_ | 642.239 | 642.2401 | 1.3 | 93.7 |
| 24 | 8.78 | Luteolin | C_15_H_10_O_6_ | 287.055 | 287.0554 | 1.2 | 95.2 |
| 25 | 8.78 | Luteoloside | C_21_H_20_O_11_ | 449.108 | 449.1086 | 1.6 | 100.0 |
| 26 | 8.78 | Scutellarin | C_21_H_18_O_12_ | 463.087 | 463.0882 | 2.3 | 99.5 |
| 27 | 8.83 | Kaempferitrin | C_27_H_30_O_14_ | 579.171 | 579.1719 | 1.8 | 100.0 |
| 28 | 9.13 | Peimine | C_27_H_45_NO_3_ | 432.347 | 432.3478 | 1.3 | 99.8 |
| 29 | 9.46 | Peiminine | C_27_H_43_NO_3_ | 430.332 | 430.3328 | 2.8 | 100.0 |
| 30 | 9.89 | Baicalin | C_21_H_18_O_11_ | 447.092 | 447.0926 | 0.9 | 100.0 |
| 31 | 10.3 | Marmesin | C_14_H_14_O_4_ | 247.096 | 247.0969 | 1.7 | 86.7 |
| 32 | 10.4 | Tiliroside | C_30_H_26_O_13_ | 595.145 | 595.1450 | 0.6 | 93.1 |
| 33 | 10.51 | Berberine | C_20_H_17_NO_4_ | 336.123 | 336.1233 | 0.8 | 85.2 |
| 34 | 11.07 | Wogonin 7-O-glucuronide | C_22_H_20_O_11_ | 461.108 | 461.1088 | 2.0 | 100.0 |
| 35 | 12.37 | Baicalein | C_15_H_10_O_5_ | 271.060 | 271.0607 | 2.2 | 88.9 |
| 36 | 13.98 | Nobiletin | C_21_H_22_O_8_ | 403.139 | 403.1395 | 1.8 | 93.8 |
| 37 | 14.48 | Schisandrin | C_24_H_32_O_7_ | 433.222 | 433.2223 | 0.5 | 97.5 |
| 38 | 15.09 | Schizandrol B | C_23_H_28_O_7_ | 417.191 | 417.1915 | 1.7 | 89.7 |
| 39 | 16.51 | Schisanhenol | C_23_H_30_O_6_ | 403.212 | 403.2123 | 1.9 | 84.8 |
| 40 | 16.57 | Schisantherin A | C_30_H_32_O_9_ | 537.212 | 537.2105 | -2.6 | 89.6 |
| Negative ion mode | | | | | | | |
| 1 | 1.22 | Quinic acid | C_7_H_12_O_6_ | 191.056 | 191.0559 | -1.0 | 78.0 |
| 2 | 1.26 | Maltopentaose | C_30_H_52_O_26_ | 827.267 | 827.2674 | 0.0 | 93.4 |
| 3 | 1.3 | L-Malic acid | C_4_H_6_O_5_ | 133.014 | 133.0144 | 0.8 | 82.8 |
| 4 | 1.54 | Shikimic acid | C_7_H_10_O_5_ | 173.046 | 173.0457 | 0.8 | 86.9 |
| 5 | 1.97 | Citric acid | C_6_H_8_O_7_ | 191.020 | 191.0199 | 1.0 | 96.0 |
| 6 | 2.54 | Succinic acid | C_4_H_6_O_4_ | 117.019 | 117.0194 | 1.0 | 83.5 |
| 7 | 3.44 | Gallic acid | C_7_H_6_O_5_ | 169.014 | 169.0143 | 0.3 | 91.7 |
| 8 | 4.1 | Guanosine | C_10_H_13_N_5_O_5_ | 282.084 | 282.0842 | -0.5 | 95.9 |
| 9 | 4.82 | Phenprobamate | C_9_H_11_NO_2_ | 164.072 | 164.0718 | 0.4 | 94.5 |
| 10 | 5.31 | Protocatechuic acid | C_7_H_6_O_4_ | 153.019 | 153.0195 | 0.8 | 95.9 |
| 11 | 5.32 | Geniposidic acid | C_16_H_22_O_10_ | 373.114 | 373.1144 | 1.0 | 94.8 |
| 12 | 5.75 | p-Coumaric acid | C_9_H_8_O_3_ | 163.040 | 163.0402 | 0.7 | 84.0 |
| 13 | 5.95 | Corilagin | C_27_H_22_O_18_ | 633.073 | 633.0739 | 0.8 | 94.0 |
| 14 | 6.29 | Protocatechuic Aldehyde | C_7_H_6_O_3_ | 137.024 | 137.0245 | 0.3 | 97.3 |
| 15 | 6.49 | Epicatechin | C_15_H_14_O_6_ | 289.072 | 289.0718 | 0.2 | 87.7 |
| 16 | 6.59 | Chlorogenic acid | C_16_H_18_O_9_ | 353.088 | 353.0879 | 0.4 | 97.9 |
| 17 | 6.88 | Esculetin | C_9_H_6_O_4_ | 177.019 | 177.0195 | 0.8 | 79.2 |
| 18 | 6.93 | Isovanillin | C_8_H_8_O_3_ | 151.040 | 151.0401 | 0.0 | 75.3 |
| 19 | 6.94 | Caffeic acid | C_9_H_8_O_4_ | 179.035 | 179.0350 | 0.2 | 86.0 |
| 20 | 7.27 | Pinoresinol Diglucoside | C_32_H_42_O_16_COOH^-^ | 727.245 | 727.2453 | -0.3 | 82.2 |
| 21 | 7.58 | Eleutheroside E | C_34_H_46_O_18_COOH^-^ | 787.267 | 787.2669 | 0.3 | 98.3 |
| 22 | 7.97 | Rutin | C_27_H_30_O_16_ | 609.146 | 609.1467 | 1.0 | 97.0 |
| 23 | 8.02 | Ellagic Acid | C_14_H_6_O_8_ | 300.999 | 300.9995 | 1.8 | 100.0 |
| 24 | 8.17 | Hyperin | C_21_H_20_O_12_ | 463.088 | 463.0888 | 1.3 | 99.5 |
| 25 | 8.49 | Aempferol-3-O-rutinoside | C_27_H_30_O_15_ | 593.151 | 593.1519 | 1.3 | 98.2 |
| 26 | 8.57 | Nodakenin | C_20_H_24_O_9_COOH^-^ | 453.140 | 453.1406 | 0.9 | 97.2 |
| 27 | 8.62 | Isochlorogenic acid A | C_25_H_24_O_12_ | 515.119 | 515.1200 | 0.9 | 92.0 |
| 28 | 8.62 | Acteoside | C_29_H_36_O_15_ | 623.198 | 623.1987 | 0.9 | 98.1 |
| 29 | 8.77 | Quercitrin | C_21_H_20_O_11_ | 447.093 | 447.0938 | 1.2 | 100.0 |
| 30 | 8.78 | Scutellarin | C_21_H_18_O_12_ | 461.073 | 461.0730 | 0.9 | 87.6 |
| 31 | 8.79 | Pinoresinol-glucoside | C_26_H_32_O_11_ | 519.187 | 519.1875 | 0.5 | 74.9 |
| 32 | 9.89 | Baicalin | C_21_H_18_O_11_ | 445.078 | 445.0780 | 0.9 | 99.5 |
| 33 | 10.4 | Tiliroside | C_30_H_26_O_13_ | 593.130 | 593.1305 | 0.7 | 77.7 |
| 34 | 10.59 | Quercetin | C_15_H_10_O_7_ | 301.035 | 301.0357 | 1.1 | 93.2 |
| 35 | 11.07 | Wogonin 7-O-glucuronide | C_22_H_20_O_11_ | 459.093 | 459.0934 | 0.2 | 98.4 |
| 36 | 11.45 | Kirenol | C_20_H_34_O_4_COOH^-^ | 383.244 | 383.2441 | 0.5 | 100.0 |
| 37 | 11.59 | Naringenin | C_15_H_12_O_5_ | 271.061 | 271.0612 | 0.1 | 92.5 |
| 38 | 13.81 | Madecassic acid | C_30_H_48_O_6_ | 503.338 | 503.3380 | 0.3 | 92.9 |
| 39 | 14.15 | Chrysosplenetin B | C_19_H_18_O_8_ | 373.093 | 373.0928 | -0.3 | 88.3 |
| 40 | 16.59 | Gingerglycolipid B | C_33_H_58_O_14_COOH^-^ | 723.381 | 723.3815 | 0.9 | 94.5 |
| 41 | 16.77 | Corosolic acid | C_30_H_48_O_4_ | 471.348 | 471.3473 | -1.5 | 100.0 |
